# Incorporating social determinants of health into agent-based models of HIV transmission: methodological challenges and future directions

**DOI:** 10.3389/fepid.2025.1533119

**Published:** 2025-02-27

**Authors:** Anna L. Hotton, Pedro Nascimento de Lima, Arindam Fadikar, Nicholson T. Collier, Aditya S. Khanna, Darnell N. Motley, Eric Tatara, Sara Rimer, Ellen Almirol, Harold A. Pollack, John A. Schneider, Robert J. Lempert, Jonathan Ozik

**Affiliations:** ^1^ Department of Medicine, University of Chicago, Chicago, IL, United States; ^2^ Engineering & Applied Sciences, RAND, Arlington, VA, United States; ^3^ Consortium for Advanced Science and Engineering, University of Chicago, Chicago, IL, United States; ^4^ Department of Behavioral and Social Sciences, Brown University School of Public Health, Providence, RI, United States; ^5^ Crown Family School of Social Work, Policy, and Practice, University of Chicago, Chicago, IL, United States; ^6^ Department of Public Health Sciences, University of Chicago, Chicago, IL, United States; ^7^ Pardee RAND Graduate School, RAND, Santa Monica, CA, United States; ^8^ Frederick S. Pardee Center for Longer Range Global Policy and the Future Human Condition, RAND, Santa Monica, CA, United States

**Keywords:** social determinants of health, agent-based models, HIV, infectious disease modeling, network models, uncertainty quantification, surrogate models, robust decision-making

## Abstract

There is much focus in the field of HIV prevention research on understanding the impact of social determinants of health (e.g., housing, employment, incarceration) on HIV transmission and developing interventions to address underlying structural drivers of HIV risk. However, such interventions are resource-intensive and logistically challenging, and their evaluation is often limited by small sample sizes and short duration of follow-up. Because they allow for both detailed and large-scale simulations of counterfactual experiments, agent-based models (ABMs) can demonstrate the potential impact of combinations of interventions that may otherwise be infeasible to evaluate in empirical settings and help plan for efficient use of public health resources. There is a need for computational models that are sufficiently realistic to allow for evaluation of interventions that address socio-structural drivers of HIV transmission, though most HIV models to date have focused on more proximal influences on transmission dynamics. Modeling the complex social causes of infectious diseases is particularly challenging due to the complexity of the relationships and limitations in the measurement and quantification of causal relationships linking social determinants of health to HIV risk. Uncertainty exists in the magnitude and direction of associations among the variables used to parameterize the models, the representation of sexual transmission networks, and the model structure (i.e. the causal pathways representing the system of HIV transmission) itself. This paper will review the state of the literature on incorporating social determinants of health into epidemiological models of HIV transmission. Using examples from our ongoing work, we will discuss Uncertainty Quantification and Robust Decision Making methods to address some of the above-mentioned challenges and suggest directions for future methodological work in this area.

## 1 Introduction

In the United States, HIV disproportionately impacts sexual and gender minorities and other marginalized communities, with the greatest impact on those with fewer resources and limited access to prevention and treatment services (1, 2). It is now well recognized that the elimination of HIV will require a greater focus on communities that experience complex and co-occurring socio-structural barriers to HIV prevention and care (3, 4). Thus, plans to eliminate HIV have increasingly focused on factors that indirectly impact HIV transmission, including housing, employment, and incarceration, which also disproportionately affect people vulnerable to HIV (3, 5, 6). However, interventions to address social determinants of health (SDOH) can be resource intensive, logistically challenging, and require long-term follow-up, making it difficult to assess their impact using standard study designs. Although not necessarily unique to interventions addressing SDOH, it may be unethical or impossible to randomly assign people to a control condition. In addition, those with the greatest need for intensified interventions may have limited interaction with traditional research settings due to the same life challenges that make it difficult to engage in HIV prevention and care. Thus, while housing and employment interventions have been developed, (7–10), they are difficult to conduct and evaluate on a larger scale.

Simulation approaches can be useful for elucidating mechanisms or evaluating interventions that are difficult to test empirically. Computational models have long been used to understand the spread of infectious diseases and to make predictions about the course of epidemics. Commonly used methods for modeling HIV transmission dynamics include compartmental models (11), such as the classic susceptible-infected-recovered (SIR) model (12) and its extensions (13), individual-level microsimulation models, including agent-based models (ABMs), and network models (14–16), which can be used in combination with ABMs (17). These models differ in their complexity, granularity, representation of mixing between populations, and behavioral impacts on disease transmission (18). Compartmental models use differential equations to model the rate of transition between compartments, assuming homogeneous populations within compartments and homogeneous mixing within and between compartments (19). ABMs, in contrast, simulate individual interactions, decisions, and behaviors using a bottom-up approach in which decision rules are applied such that the interactions between agents and their behaviors can give rise to population-level patterns not explicitly programmed into the model (20, 21). One of the benefits of ABMs as compared to compartmental models for modeling HIV transmission dynamics is the ability of ABMs to explicitly model heterogeneous individual agent characteristics, behaviors, and interactions among agents that result in HIV transmission. Network models focus on detailed representation of networks that connect individuals and through which information or disease can spread (14, 16).

Whereas classic SIR models do not incorporate behavioral dynamics, recent work has demonstrated the importance of behavioral responses in epidemic modeling (18, 22, 23), where people change their behaviors in response disease dynamics, which in turn affects transmission, resulting in behavioral-epidemiological feedback (22, 24). Decisions about whether to adhere to pharmaceutical or non-pharmaceutical interventions (NPI) are likely to be shaped by the prevalence and severity of the disease, perceived risk, and the cost of adhering to the interventions, including social or economic costs (24). These decisions can change over time in response to the epidemic and can be shaped by social or contextual factors that affect the ease of adherence or access to NPI or vaccinations.

ABMs simulate individual agent decision making, making them well suited to explicitly model behavioral-epidemiological feedback and to observe emergent patterns resulting from this feedback over time (21). For example, individuals may change their adherence or use of PrEP in response to changes in the HIV epidemic and perceived risk, and SDOH can affect the ease with which individuals change their behavior in response to disease dynamics. ABMs can also be used to conduct counterfactual experiments in which candidate interventions are systematically evaluated and compared. This can facilitate identification of effects that would be difficult to isolate using traditional statistical approaches (25, 26) and more efficient and focused intervention development (27, 28). In addition to allowing for behavioral feedback, ABMs are well suited to incorporate the dependency structures and feedback loops common among co-occurring social determinants of health (21, 26, 29). Thus, ABMs can demonstrate the potential impact of combinations of interventions that are not typically feasible to evaluate in empirical settings. Applications of ABMs for studying the impact of complex social phenomena on health outcomes have been described, most notably in the context of chronic diseases, where ABMs have elucidated network and system level drivers of obesity (30), tobacco use (31) and violence and PTSD (32–34). However, most ABMs of HIV transmission to date have focused on more proximal influences on transmission dynamics (e.g., sexual behaviors, networks) (17, 35, 36), and do not include distal factors that may impact transmission indirectly. This is in part due to the complexity of the relationships among SDOH and HIV and limitations in the measurement and quantification of causal relationships among SDOH.

ABMs are typically based on the inherent assumption that associations among the variables used to parameterize the model reflect causal relationships, but causality often cannot be inferred from empirical data. Although one of the benefits of ABMs is their ability to combine and triangulate information from multiple sources, it can be difficult to reconcile variation in estimates across different data sources. Effect sizes often vary widely, obtained from observational studies conducted across a wide range of populations and time periods. Uncertainty exists in terms of the magnitude and direction of associations in the variables used to parameterize the model, as well as in the model structure itself (i.e., the causal relationships among the variables being modeled). Thus, while a primary motivation to use ABMs is often that they offer the opportunity to study aspects of complex systems that are difficult or impossible to study empirically, ABMs require detailed empirical data or strong assumptions to draw conclusions about the systems they represent. These issues are not unique to modeling SDOH, but they are likely to be particularly relevant since data on SDOH and interventions to address them are often limited and complex social constructs are difficult to accurately measure even when data are available. Understanding how uncertainty and bias in empirical data impact ABM outputs, and how ABMs can be used to guide public health decision-making in the presence of this uncertainty, are active and fruitful areas for future work.

Recent reviews have described various approaches to modeling social determinants of health in the context of HIV, including statistical and compartmental models and ABMs. Hogan et al. (37) discuss uses of mechanistic and statistical models to answer different types of questions related to SDOH in HIV. De Oliveira et al. (38) describe the process of developing a conceptual framework for incorporating SDOH into a compartmental model of HIV. ABMs are valuable for studying the impact of human behavior on HIV because they allow explicit modeling of heterogeneous agent behaviors and interactions, but with a significant increase in complexity and computational resources compared to compartmental and statistical models. The sections below focus on ABMs because of their utility for modeling heterogeneous individual behaviors and transmission networks, and the impact of SDOH on agent behaviors, interactions, and disease dynamics. We also discuss challenges unique to ABMs compared to other types of models.

The remainder of the manuscript is organized as follows. First, we describe current examples of how SDOH can be incorporated into agent-based models of HIV transmission. We then describe approaches to addressing uncertainty and their limitations. Finally, we discuss ongoing applied work in the use of ABMs to support public health decision making in the presence of deep uncertainty.

## 2 Approaches to incorporating SDOH into agent-based models

### 2.1 Developing a conceptual model

The first step of incorporating SDOH into the ABM is to develop a conceptual framework for describing how SDOH impact HIV transmission. Embedded in this framework are a number of causal hypotheses about how various factors relate to one another. These hypotheses are then translated into the ABM. Based on our previous research, review of the literature, feedback from community members, and analysis of empirical data, we hypothesized that SDOH impact HIV transmission through one or more of the following mechanisms: (1) changes in individual psychosocial factors (e.g., substance use, mental health) that impact transmission related behaviors, prevention and care engagement, and medication adherence; (2) changes in sexual networks that affect the probability of serodiscordant sexual partnerships; (3) changes in health care access or engagement (e.g., as a result of employment and insurance changes, economic hardship, or housing instability) that impact the probability that individuals are retained in HIV care or on PrEP. From our conceptual model, we focus on modifiable or actionable intervention levers. For example, we do not model racism explicitly, but rather the model incorporates the effects of racism, discrimination, and other larger social constructs on factors that impact HIV transmission; i.e., housing, employment, and incarceration can affect use of ART or PrEP, which impact the probability of HIV transmission.

### 2.2 Incorporating relevant pathways and processes to model interventions to address SDOH

**Figure 1:**
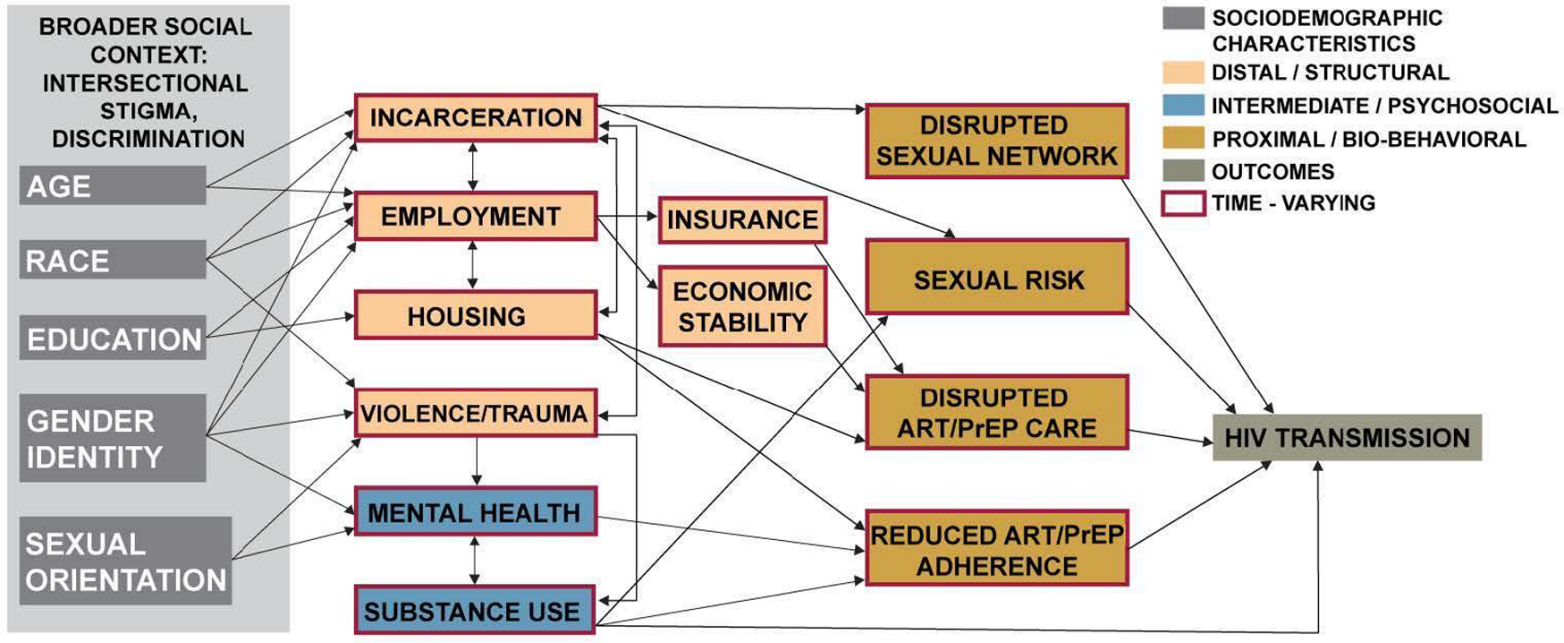
Conceptual model of the pathways by which social determinants of health impact HIV transmission.

**Figure 2:**
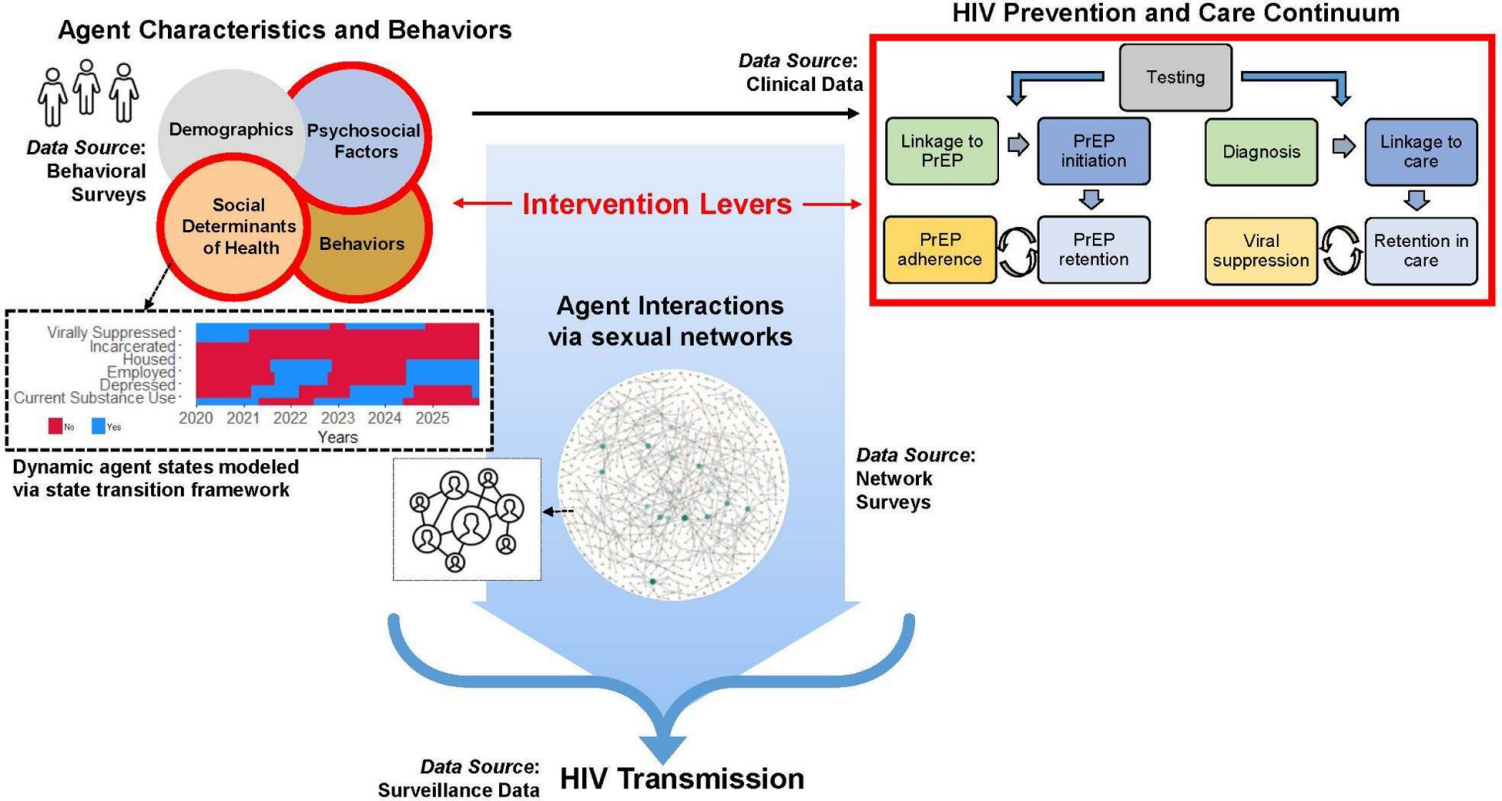
Components of the integrated framework for modeling social determinants of HIV (INFORM-HIV).

To evaluate the potential impact of interventions to address SDOH on HIV transmission, we developed the Integrated Framework for Modeling Social Determinants of HIV (INFORM-HIV) ABM. The model extends and expands an agent-based network model (17) of HIV that incorporates sexual behaviors and networks, HIV testing, diagnosis, linkage to care, and ART and PrEP use (Figure 1). The original model was modified by (1) specifying hypothesized mechanisms by which SDOH impact HIV prevention and care engagement directly and indirectly via intermediate factors and assigning rules by which these factors impact model processes, and (2) incorporating agent characteristics that impact the probability of experiencing incarceration, housing instability, and unemployment while accounting for correlations among agent attributes. A simplified model diagram is shown in Figure 2. Agent characteristics are assigned probabilistically at the beginning of the simulation to match the empirical distribution of SDOH in the population represented in the model and the correlations among SDOH. SDOH can produce changes in one or more domains, and relationships among factors within and between domains can be cyclical and bidirectional. As the simulation progresses, agents make decisions at various steps in the HIV prevention and care continuum (e.g., whether to get tested for HIV, take medication, adhere to PrEP/ART). Decision probabilities reflect agents’ past and current experiences of housing instability, employment, and other structural factors, which can change in response to internal or external factors, including substance use. Agent decisions are also influenced by individual psychosocial factors, such as mental health. Structural and psychosocial factors can influence each other and can vary over time.

### 2.3 State transition models

In order to allow for dynamic (i.e., time-varying) agent behaviors, states, and characteristics, we apply a state chart approach within the ABM. Statecharts are a type of state diagram (39), or visual depiction of sequential control procedures or algorithms. Early examples of state diagrams include Moore machines (40) and Mealy machines (41). Our implementation is based the Repast Simphony implementation of Harel’s statecharts (42) described in (43).

Each agent has multiple state charts, each tracking separate attributes, such as housing or employment, with distinct states (e.g., housed/unhoused, or employed/unemployed). Transition probabilities determine when an agent moves from one state to another. The transitions incorporate dependency among different agent attributes, such that rates of transition between states in one statechart can be based on the occupied states of another statechart, as well as on any other agent attribute. Transitioning between two states is determined by a probability where transitions are more or less likely depending on agent characteristics and previous states. We applied a multiplier to base transition probability to indicate the increased or decreased probability of transitioning into or out of a state based on other agent characteristics. For example, an agent is more likely to transition from housed to unhoused at a subsequent time step if they become unemployed than if they remain employed.

## 3 Modeling transmission networks

Network models provide a statistically principled approach to representing concurrency (i.e., having one or more sexual partnerships that overlap in time as opposed to serial or sequential partnerships that do not overlap), which is thought to be an important contributor to HIV transmission dynamics in some settings due to efficiency of transmission during the acute phase of infection (44–46). Extensive methodological and software development has been undertaken in modeling networks in the context of infectious diseases, and we refer the readers to the published literature for in-depth reviews and example applications (47–49). Below is a brief overview of how HIV transmission networks are modeled within the ABM using exponential-family random graph models (ERGMs).

### 3.1 Exponential-family random graph models (ERGMs)

ERGMs are a class of statistical models used to represent the structure of social or sexual networks through the presence or absence of ties, which are assigned to approximate target statistics describing the observed network (50). Target statistics reflect the likelihood that agents form partnerships based on various individual (nodal) or dyadic (edge) characteristics. For example, SDOH can impact the likelihood of partnership formation and dissolution, this impacting the network structure. Extensions of ERGMs, separable temporal exponential-family random graph models (STERGMs), are used to represent dynamic networks over time (51). These models are said to be “separable” because the factors affecting partnership formation and dissolution are assumed to be independent conditional on the existing network structure (16). Thus, STERGMs have separate formulas for partnership formation and dissolution. While the formation and dissolution models are conditionally independent at a given time step, they are modeled jointly over time, allowing the structure of the tie formation model to be identified in the context of the existing persistence model (16). To optimize the computational cost of fitting ERGMs, we use the recommended “statnet” approximation for cases where the networks are relatively sparse (such as in sexual networks) and the relationships are of at least moderate duration (relative to the time units of the model) (52).

Main and casual partnership networks are estimated using the “ergm” package in R (47), and the coefficients for the ERGM parameters are estimated using Markov Chain Monte Carlo methods (47, 48, 53), using Carnegie et al.’s approximation method (52). These estimated adjusted coefficients are used to simulate main and casual networks, which are read into the ABM. The “rpy2” package (54) is used to connect the Python code to the ERG model in R.

### 3.2 Uncertainty in network structure

ERGMs are typically developed to approximate target statistics, and the formation and dissolution coefficients are used to simulate networks dynamically over time. ERGMs seek to maintain equilibrium with respect to the target statistics, and the amount of variation across stochastic realizations of a network given a set of formation and dissolution parameters is often not characterized. Because HIV is transmitted via sexual networks, the extent to which the network structure (e.g., in terms of mixing patterns, partnership duration, concurrency, etc.) accurately reflects the underlying network in the population represented in the model is essential for accurately modeling HIV transmission. Computational demands of modeling networks has previously limited the extent to which sensitivity analyses involving varying network structures could be conducted. However, with increases in computational resources, it is possible to experiment with different network configurations to determine the impact of the network structure on model outputs. Such analyses are important given the challenges involved in measuring networks using empirical surveys (55–57). Additionally, there may be limited data on how SDOH impact network structure and dynamics.

## 4 Methodological considerations for empirical data: obtaining causal effect estimates

Associations have been demonstrated between various SDOH (e.g., housing instability, incarceration, economic hardship) and HIV risk (58, 59), mental health and substance use (60, 61), and HIV prevention and care engagement (61–63) across multiple geographic contexts and populations. However, much of the existing evidence base has come from cross-sectional studies or lacked power to test for interactions or mediation effects. Further, many of these relationships are likely cyclical (e.g., housing instability creates employment barriers which leads to ongoing lack of resources and reduced ability to obtain stable housing). This makes it challenging to determine directionality in terms of the causal structures underlying the model, which is necessary to understand which interventions or combinations of interventions would have the greatest public health impact.

Ideally, for each factor of interest (X) and outcome (Y), it would be possible to determine (1) whether X affects Y (with the assumption that the effect is causal), (2) how X affects Y (i.e., what are the mechanisms, or pathways, by which X affects Y, including direct and indirect effects), and (3) whether the effect of X on Y is the same across the whole population being modeled, or whether it varies across subgroups of the population (i.e., is there effect heterogeneity). If all of the above statements are known, we can use the model to answer questions such as “What would be the impact of intervening on X on the outcome Y?” (in terms of absolute and relative effects, cost, or any other relevant effect measure). We can also answer questions such as “Would interventions that are focused on a subgroup or subgroups of the population be more efficient (in terms of cost, infections averted, etc.) than more broadly targeted interventions?”

Many traditional applications of ABMs have focused on answering the two questions above, assuming that the preceding items (1–3 above) are known and quantified. There is also typically the assumption that model parameters and calibration targets are unbiased and measured without error (this is discussed further below in the context of sensitivity analysis and calibration). However, these assumptions often cannot be easily tested or verified for most SDOH or other factors that are not amenable to testing in randomized controlled trials. Thus, conclusions are highly dependent on the availability and quality of the data used to parameterize the model. There is thus a need to account for and address uncertainty in models seeking to understand the potential impact of interventions on SDOH for reducing HIV transmission. This is discussed in further detail in the sections that follow.

ABMs’ potential to aid epidemiological causal inference has been recognized (20, 25, 26) but applications in the context of complex social phenomena have been limited (34). Recently published work has demonstrated how uncertain or biased input parameter estimates can result in biased results from ABMs (64, 65). These illuminating examples have focused on clinical data and endpoints, and the potential for bias is likely amplified in the case of social determinants of health. Debate remains about the circumstances under which necessary assumptions can be tested and verified (66), and estimates obtained from one population can be transported to another (64, 67). More work is needed to determine to what extent uncertainties can be addressed using computational approaches and how ABMs can be used to study highly complex systems in the presence of uncertainty.

## 5 Approaches to dealing with uncertainty: uncertainty quantification, sensitivity analysis, and calibration

Understanding the magnitude and potential impact of various forms of uncertainty is essential in ABM research because it directly impacts conclusions that can be drawn from models, particularly when comparing interventions in order to make public health decisions about use of limited resources. Broadly, uncertainty is categorized into two types: aleatory and epistemic. Aleatory uncertainty captures the inherent randomness in the stochastic processes that drive agent interactions within an ABM and the empirical data being observed. Epistemic uncertainty, in contrast, arises from a lack of knowledge about the process or input to the computational model. Generally, epistemic uncertainty is considered “reducible” by collecting more data, refining model parameters, enhancing model structure, or conducting further experiments, while aleatory uncertainty is “irreducible.” The objective is to identify and account for all quantifiable uncertainties within projections created by the ABMs.

HIV transmission models that integrate the influences of SDOH are inherently complex, with many relationships that are indirect, bi-directional, or non-explicit. Such complexity introduces *structural uncertainty* due to the incomplete understanding of causal relationships among SDOH and between SDOH and HIV. This complexity necessitates the inclusion of a large number of parameters to control the various aspects of the model. However, estimating these parameters is challenging, often relying on incomplete data that may include significant measurement errors, which underscores the need for large-scale sensitivity analysis to assess the robustness of the model. Thus, the overall task becomes computationally demanding due to the model’s intricacy. Although sensitvity analysis (SA) has been applied in ABM research previously [e.g., (68)], complex models integrating SDOH entail far larger parameter spaces and, hence, more uncertainties to address.

In the following sections, we provide a brief outline of approaches for examining the impact of various forms of uncertainty on model outcomes using uncertainty quantification and sensitivity analysis tools (we refer readers to comprehensive resources such as (69) and other cited works for a more in-depth understanding). We also review how complex models with uncertainties, including ABMs that seek to understand how SDOH factors impact HIV transmission, can be calibrated.

### 5.1 Uncertainty quantification and sensitivity analysis

Uncertainty quantification (UQ) and sensitivity analysis (SA) are complementary approaches that are often performed together to understand the impact of uncertainty on model outcomes. UQ involves characterizing the uncertainty in a model’s input parameters that arise from measurement error or natural variation, often in the form of distributions or ranges (sometimes referred to as *uncertainty characterization*) and subsequently evaluating the impact of this uncertainty on model outcomes (sometimes referred to as *uncertainty propagation*) (70, 71). In other words, UQ is used to link uncertainty in the input parameters to uncertainty in the model outputs. Sensitivity analysis is then performed to understand which input parameters have the most impact on model outcomes. In an ABM, some parameter values are derived empirically from observed data, while others, which are not directly observable, are estimated through calibration and data assimilation. Uncertainty in the data can lead to varying degrees of influence on model parameter ranges and distributions. SA aids in simplifying the model by identifying parameters with minimal impact on outcomes and facilitating dimensional reduction. It also highlights parameters with large impacts, where improving on empirical precision would be beneficial to reduce model output uncertainty.

The first step in SA is to identify model parameters and define their ranges. Parameter ranges may be informed by empirical data, previous studies, expert opinion, or, when data is sparse, assigned a broad range. When there is a large parameter space, an initial screening is useful to identify parameters with minimal influence, allowing these to be fixed at specific values and thereby reducing the problem’s dimensionality. The Morris method (72) is effective for this screening, requiring only mr(p+1) simulation runs, where m is the number of settings tested per parameter p, and r is the number of replicates when a model is stochastic. This approach provides an efficient way to identify influential parameters before performing a full-scale SA. However, as model complexity, and in turn, the number of parameters, increases, comprehensive UQ/SA can become computationally infeasible (70). Surrogate models, discussed next, and high-performance computing (HPC) workflows become essential for global SA in large computational models (73–75).

### 5.2 Surrogate models

Surrogate models, also referred to as emulators or reduced-order models, serve as efficient approximations to actual models, in the current context serving as surrogates for ABMs. These are mathematical representations of the relationships between inputs and outputs of a model, and are constructed to predict outputs of a model based on a limited number of simulations. Trained surrogates can be used to quickly reproduce predictions from a model without having to run the model (76–79). Surrogates can significantly reduce the computational costs in ABM analyses, thus facilitating large-scale UQ/SA. Building a surrogate model involves selecting an appropriate functional form, such as Gaussian processes (80), polynomial chaos expansions (81), or neural networks (82), and fitting it to a set of model input/output pairs, using static or adaptive experimental designs (80). Once fitted, the surrogate provides fast approximations of ABM outputs across the input space, enabling extensive sampling needed for UQ/SA and other critical analyses such as ABM calibration (83), discussed next.

For SA applications, surrogates can be used to estimate Sobol sensitivity indices (84). To train a Gaussian process (GP) surrogate, an initial simulation campaign is performed, e.g., using a Latin hypercube space-filling design (85). If the parameter space is high-dimensional, a single round of GP training can require a significant number of simulations to produce acceptable prediction accuracy. However, by integrating the GP with an active learning strategy, such as the one developed in (86), the total simulation budget can be reduced. This approach starts with a small number of simulations and iteratively adds more, prioritizing those that enhance the estimation of Sobol sensitivity indices. The active learning framework can be combined with various GP constructs to tailor the surrogate to the specific needs of the underlying model. In addition, approximate GPs can be used to accelerate the computation of sensitivity indices by constructing surrogates that are local to specific parameter subspaces (87).

### 5.3 Calibration

Prior to scenario analyses or predictions, ABMs need to be calibrated to ensure that the outputs from the model are consistent with empirical data targets. Calibration involves running the model with different sets of parameters and comparing the output to empirical data with the goal of minimizing model error (88). Calibration targets can be evaluated based on point estimates or ranges, but the greater the uncertainty in the calibration targets the more challenging and computationally expensive the calibration becomes.

Uncertainty in calibration targets is an important issue in HIV modeling, particularly when modeling sub-populations in which the denominators defining the population size estimates used in incidence and prevalence calculations have to be estimated from multiple sources (89–91). This results in additional uncertainty in surveillance estimates above the standard limitations of surveillance data which typically suffer from some degree of under reporting and other reporting biases. Thus, calibration approaches that address uncertainty in both the input parameters and the calibration targets are needed.

#### 5.3.1 Approaches to calibration

*History matching* History matching is a common calibration technique which works by systematically ruling out parameter values that are inconsistent with the observed data. Unlike other calibration methods that seek to optimize parameters directly, history matching iteratively eliminates implausible regions of the parameter space, zooming in on parameter combinations that yield predictions aligned with empirical data (92, 93). History matching has seen its use in healthcare applications, particularly for calibrating high-dimensional models. For computationally expensive models such ABMs, it can be paired with surrogate models, enabling efficient exploration of the parameter space without exhaustive simulations (94). The iterative refinement process is especially applicable to healthcare applications, where robust model calibration is necessary for forecasting and evaluating interventions under uncertainty. By focusing computational resources on the most plausible parameter sets, history matching provides a rigorous yet computationally feasible approach to calibration, enhancing model reliability in critical healthcare applications (95).

*Approximate Bayesian computation* Approximate Bayesian computation involves sampling a set of parameters from a prior distribution and testing if simulated model outputs (*simulated targets*) are within pre-specified thresholds of empirical values (*empirical targets*). A parameter set is accepted if the error is smaller than the threshold and otherwise rejected. Testing a large number and range of parameters can be used to approximate the posterior distribution, i.e., the probability of the parameters given the data. Sampling the parameter space and evaluating model outputs can become computationally expensive so efficient sampling methods are needed. Different ABC algorithms have been applied in the literature, ranging from simple rejection sampling to sequential Monte Carlo approaches (96, 97).

*Bayesian Calibration* Bayesian calibration using GPs has become a widely adopted approach in various modeling domains, providing a principled way to calibrate complex models by accounting for both parameter uncertainty and model discrepancy. This method combines Bayesian inference with a GP-based surrogate model approximating the computationally expensive model across the input space, enabling efficient estimation of posterior distributions for model parameters (98). The Bayesian approach allows for incorporating prior knowledge and updating beliefs about model parameters based on observed data, yielding a posterior distribution that represents uncertainty about parameter values and the empirical data. This is particularly relevant in healthcare applications, such as modeling disease progression or patient outcomes, where data is often limited, error-prone, and computational resources are constrained (99). Bayesian GP calibration also facilitates robust UQ by capturing both aleatoric and epistemic uncertainty. As a result, the model predictions are accompanied by realistic uncertainty estimates, (100), which, as discussed in the following section, is important in the context of decision-making in critical healthcare scenarios.

*Optimization* Optimization-based calibration with active learning builds upon GPs to efficiently refine parameter estimates in complex simulation models. In this approach, the GP surrogate enables rapid evaluations of an otherwise computationally expensive objective function (i.e., the expensive simulation), guiding the search for optimal parameter values. Active learning further aids in this process by adaptively selecting data points in regions of high model uncertainty or near potential optima, thus prioritizing areas where new information would most improve model fidelity (101). The combination of GPs and adaptive sampling reduces the need for exhaustive simulations, focusing computational resources on the most informative parts of the parameter space (102).

The INFORM-HIV ABM contains complexities that require further considerations for applying the calibration approaches presented above. These include the need to address potential issues of non-identifiability (103). To improve feasibility there is also the need for dimension reduction techniques such as active subspaces (104) and variational autoencoders (105). These methods aim to capture lower-dimensional representations of the input data, facilitating more efficient exploration of the parameter space while accounting for the complex interdependent relationships represented in INFORM-HIV. Another challenge is addressing multiple (potentially dependent) calibration targets. A GP can be extended to a multi-output framework, where a single set of input parameters affects several outputs, and the correlation between these outputs is modeled via a covariance function (106). Alternatively, multi-objective optimization provides a method for optimizing multiple, often conflicting, outputs. This approach aims to identify a set of optimal solutions, known as the Pareto front, where improving one objective would degrade another. GP surrogates can be integrated into this optimization framework to enable efficient solution exploration (107).

## 6 From models to decision making

Despite the uncertainties described above, public health stakeholders must make decisions in the presence of limited information. Developing solutions to complex public health problems like HIV involves collaboration among many stakeholders from different sectors, with different and often competing priorities, and differences in focus in terms of policies, intervention levers, and measures of success. Incorporation of the extensive knowledge and applied public health experience among stakeholders is essential for developing meaningful models for public health decision making. However, there is a challenge in translating this knowledge into models that can inform decision making in contexts where there is a high degree of uncertainty from a data perspective as discussed in Section 5, but also characterized by deep uncertainty. In other words, in addition to uncertainties in the data used to parameterize models, there are aspects of the current and future states of the world that are unknowable. Tools to help stakeholders make informed decisions in the presence of uncertainties in data, causal structures, transmission networks, and future states are needed to develop effective tools for modeling interventions to address SDOH in complex systems such as HIV.

*Robust Decision Making (RDM)* is a multi-scenario, multi-objective decision-analytic approach designed to inform policy decisions when decision-makers and scientists lack confidence or agreement in either (i) the appropriate models to describe interactions among a system’s variables, (ii) the probability distributions to represent uncertainty about key parameters in a model, or (iii) how to value the desirability of alternative outcomes (108). Under these conditions, often labeled as *deep uncertainty* (109), traditional decision-analytical approaches can lead to gridlock over contested assumptions or unwarranted overconfidence in model predictions, both of which can prove counterproductive (108).

RDM offers a rigorous approach to address the deep uncertainties inherent in modeling SDOH and their impacts on HIV transmission described in this paper. The RDM process typically begins with stakeholder engagement to develop a conceptual framework, identify potential interventions, set policy objectives, and recognize key uncertainties. These elements are then programmed into a simulation model, such as an agent-based model. Experiments are conducted to test how different interventions perform across a wide range of future scenarios and assumptions. RDM studies then focus on assessing the robustness of policy decisions to uncertainty and on clarifying the conditions under which policies fail to meet policymakers’ goals. Analysis of the results focuses on identifying robust strategies that perform well across many plausible futures rather than optimizing for a single set of assumptions (110, 111). RDM’s focus on clarifying the conditions under which policymakers’ goals can be achieved is particularly relevant for HIV elimination efforts as policymakers seek combinations of policy interventions that can achieve HIV elimination goals.

Existing RDM applications in the life sciences provide examples of how RDM can help address deep uncertainty in HIV modeling. For instance, (112) used a scenario discovery approach to identify conditions under which blood-based tests could be superior to established tests for colorectal cancer screening. This approach identified regions in the parameter space where blood tests could be more cost-effective than existing tests. Similarly, RDM was used to inform the decision to reopen the economy following the vaccination campaigns in the COVID-19 pandemic, addressing uncertainties about the transmissibility of future variant strains and the population’s willingness to accept a new vaccine (113, 114). Following the COVID-19 pandemic, RDM was also used to demonstrate how public health decision-makers can weigh health and economic outcomes while accounting for uncertainty when introducing nonpharmaceutical interventions (115).

RDM can support HIV elimination decision-making in multiple ways. First, RDM can be used to stress-test the pathway to HIV elimination that current HIV elimination plans aim to achieve. Such stress-tests can be helpful to increase the confidence that existing HIV elimination plans can achieve HIV elimination goals, or may reveal that current levels of funding and effort are not sufficient to achieve aspirational goals. Although advocates and policymakers have set goals to mobilize resources, it is unclear whether those goals are either overambitious or are not sufficient to achieve HIV elimination. Second, RDM can be used to stress-test the robustness of *multiple alternative* policy portfolios to help decision-makers understand how to “dose” different policy interventions. Finally, in an environment where funding for HIV elimination interventions cannot be taken for granted, RDM can help decision-makers understand the critical policies without which HIV elimination cannot be achieved.

INFORM-HIV could be further extended to include endogenous adaptation in agent behaviors in response to epidemic trends. Currently, rules by which agent characteristics and interactions impact epidemic dynamics are specified as exogenous inputs. However, in the context of HIV and other infectious diseases, people often adapt their behavior in response to changes in the epidemiology of the disease, in addition to factors such as the availability of effective treatments or vaccines and perceived risk (22–24). Adaptive responses could also be modified by SDOH. RDM tools could play a critical role given the additional deep uncertainties associated with modeling adaptive behavioral responses.

## 7 Conclusions and future directions

When addressing complex public health issues such as HIV, policymakers are often required to make decisions in the presence of deep uncertainty in multiple domains. ABMs can help to understand and quantify the population-level impacts of combinations of biomedical, psychosocial, and structural interventions. However, to quantify the impact of these interventions, ABMs require parameter estimates that reflect causal relationships and structures among many interconnected factors, which are hard to quantify using standard statistical techniques. There is a need for further development of computational methods to address uncertainty in highly complex ABMs, as well as tools to aid in public health decision making in order to translate ABM results into actionable interventions. Integrating methods from statistics, agent-based modeling, and robust decision-making could offer new tools for understanding and addressing complex public health issues.
